# Effect of miR-27b on the proliferation and apoptosis of diffuse large b-cell lymphoma cells by targeting the regulation of MET/PI3K/AKT pathway

**DOI:** 10.1007/s12672-022-00589-9

**Published:** 2022-12-11

**Authors:** Rui Zhang, Tianjiao Huang, Jinfeng Li, Hong Zhou, Xuemei Wang

**Affiliations:** Department of Hematology, The Second Affiliated Hospital of Qiqihar Medical College, No. 37 Zhonghua West Road, Jianhua District, Qiqihar, 161006 Heilongjiang Province China

**Keywords:** Diffuse large B-cell lymphoma, miR-27b, PI3K, AKT pathway, Apoptosis

## Abstract

**Background:**

This study aimed to explore the regulation of miR-27b expression on MET/PI3K/AKT pathway, and to explain its effect on biological functions of DLBCL cells.

**Methods:**

The expressions of miR-27b and MET gene in DLBCL cells and normal human B cell lines were determined by qRT-PCR. miR-27b expression in DLBCL cell line Toledo was over-expressed with the cell transfection method. The proliferation of DLBCL cells was determined by MTT. And the invasiveness of DLBCL cells was determined by Transwell. The level of apoptosis in DLBCL cells was determined by ELISA. miR-27b targeting of MET was verified by dual- luciferase reporter assay. The activation of MET/PI3K/AKT pathway and the expression of downstream related proteins were determined by Western blot.

**Results:**

The results showed that miR-27b was poorly expressed in DLBCL cell lines compared with normal human B cell lines, and was associated with its high proliferation, high invasiveness and low apoptosis level. High miR-27b expression can reduce the proliferation and increase the apoptosis level in DLBCL cells. By examining the effect of miR-27b over-expression on the MET/PI3K/AKT pathway, it was found that miR-27b can inhibit the proliferation and invasiveness and promote the apoptosis of DLBCL cells by targeting the inhibition of MET expression and the activation of PI3K/AKT pathway.

**Conclusion:**

miR-27b can inhibit the proliferation and invasiveness of DLBCL cells and promote the apoptosis of the cells by targeting MET/PI3K/AKT pathway.

## Highlights

1. MET is a direct target of miR-27b.
2. Low expression of miR-27b is correlated with the growth activity of DLBCL cells.
3. miR-27b inhibits the proliferation and invasiveness of DLBCL cells, and promotes the apoptosis of the cells by targeting MET and inhibiting the activity of the MET/PI3K/AKT pathway.

## Introduction

Diffuse large B-cell lymphoma (DLBCL) is a heterogeneous and invasive lymphoma that is currently the most common type of adult non-Hodgkin’s lymphoma [1]. Its pathogenesis, clinicopathological features and high invasiveness are closely correlated with genetic abnormalities [2]. Recently, R-CHOP chemotherapy regimen has become the first-line treatment regimen for DLBCL due to the improved long-term survival rate of patients with DLBCL [3]. However, more than 20% of patients with DLBCL still have the characteristics of recurrence or showing primary refractoriness after receiving regular treatments [4]. Over these years, several other regimens have also been adopted in the clinic to improve the long-term survival rate of patients [5], which, however, were not effective.

To accurately identify high-risk patients with DLBCL, investigators developed different stratification tools such as International Prognostic Index (IPI) [6], Gene expression profiling (GEP) and immunohistochemical analysis, etc. [7, 8]. However, existing analytical tools cannot accurately identify high-risk patients with DLBCL who have been treated ineffectively, and new more effective biomarkers are needed to better stratify the risks in patients and guide subsequent treatments. Crizotinib, an inhibitor of tyrosine kinase receptors [9], has been found to be effective in non-small cell lung cancer [10] and has shown some efficacy in the treatment of DLBCL. But due to the strong resistance and recurrence using crizotinib [11], patients often have short period of remission. So more targeted diagnosis and treatment methods are needed.

Therefore, microRNA has been paid more attention to and studied by more and more researchers in recent years as a new biomarker that may predict the treatment effect and prognosis in patients with DLBCL. microRNA is a small non-coding RNA. It exerts oncogenic or tumor suppressive effects by binding to the 3′untranslated region (UTR) of target mRNAs to up-regulate the activity of their corresponding targeted genes and related pathways at the post-transcriptional level, which is of great significance in the diagnosis, classification and prognosis prediction of various cancers [12–14]. For example, studies have shown that microRNA LPAR3 can upregulate MET gene expression and activate the PI3K/Akt pathway, promoting the migration, invasion and metastasis of esophageal squamous cell carcinoma (ESCC) cells in vivo and in vitro [15]. miR-1-3p, miR-23b-3p, miR-34a-5p and miR-130a-3p all have conserved binding sites to MET and can influence the progression of pancreatic ductal adenocarcinoma (PDAC) by targeting MET through the PI3K/AKT signaling pathway [16]. microRNA-27b is a kind of microRNA that regulates the proliferation, differentiation function and apoptosis of human cells. It mainly regulates the growth cycle and division proliferation by regulating target genes such as Nischarin, FZD7 and PPARγ. It is often regarded as a tumor suppressor. However, some recent literature also reported that miR-27b may play a key role in the development of DLBCL [17]. However, its clinical significance and potential mechanism in this process are not completely clear and need to be further explored.

MET is a proto-oncogene that exerts its functions in regulating the growth cycle, proliferation, migration and invasiveness of cells by modulating signaling pathways in cells. Previous studies have confirmed that MET expression is active in various cells of tumors, such as lung cancer, brain cancer and glioma, which is closely related to the growth and proliferation of tumor cells [18–20]. PI3K/AKT signaling pathway is one of the main cell signaling pathways, which plays an important role in regulating the proliferation, growth, size, metabolism and motility of cells. PI3K/AKT pathway has been confirmed by multiple studies in the development of multiple human cancers, and several clinical trials have confirmed that inhibiting the activity of PI3K/AKT pathway can inhibit the proliferation of tumor cells [21].

## Methods

### Target prediction

By querying the Targetscan database, MET was predicted to be a potential downstream target of miR-27b. (http://www.targetscan.org/cgi-bin/targetscan/vert_71/view_gene.cgi?rs=ENST00000397752.3&taxid=9606&members=&showcnc=0&shownc=0&showncf1=&showncf2=&subset=1#miR-27-3p).

### Cells and cell lines

Twenty biopsy pathological samples were collected during surgery from patients diagnosed as DLBCL with complete prognosis information at the Second Affiliated Hospital of Qiqihar College of Medicine from January 2020 to December 2021. None of the patients had heart, brain, kidney, or other vital organ disease, other chronic liver disease, or a disease that could cause metabolic disorders. All patients underwent surgery and were pathologically diagnosed with diffuse large B-cell lymphoma with no signs of distant metastasis. No patient received any form of medication and radiotherapy and/or chemotherapy prior to surgery.

Toledo DLBLC cell lines were purchased from Shanghai Qida Biotechnology Co., Ltd. and normal human peripheral B cell lines were purchased from IPHASE. The frozen DLBLC cell lines and normal human peripheral blood B cell lines were thawed in a constant water bath at 37 °C. The dissolved cell suspension was added to the centrifuge tube with a pipette gun, which was then injected with 10% inactivated FCS medium at 1000 rpm for 5 min, and the supernatant was discarded. Cell suspension was prepared by adding 1 mL of medium and inoculated into sterile culture flask containing 5%CO_2_ and incubated at 37 °C for 24 h.

### Cell transfection

DLBCL cells were inoculated in 6-well plates, supplemented with 500 μL of Lipofectamine 2000 (Thermo Fisher Scientific) in 1.5 mL serum-free medium. DLBCL cells were incubated in 5% CO2 at 37 °C for 6 h. Then, the culture medium in each well was replaced with normal media containing serum, which was then cultured for another 24 h before the following test step was performed. Transfected plasmids (miR-27b mimics and miR-27b NC) were provided by GenePharma (Shanghai, China). Crizotinib (MET inhibitor) was provided by InvivoChem.

### RNA extraction and qPCR amplification

DLBCL cells lysed with 1 mL TRIzol (Invitrogen; Thermo Fisher Scientific, Inc.) were thoroughly mixed with chloroform. According to the instructions of kits, 10 ug RNA sample was reversely transcribed into cDNA with SuperScript III reverse transcriptase and TaqMan MicroRNA reverse transcription kits (Thermo Fisher Scientific, Inc.). qRT-PCR was performed with ABI 7500 Real-Time PCR (Applied Biosystems; Thermo Fisher Scientific, Inc), and microRNA-27b concentration was recorded and graphed. The total reaction system was 10 μL. qRT-PCR parameters were: 95 °C denaturation for 30 s, and 60 °C annealing for 40 s, for a total of 36 cycles [22].

### Detection of cell proliferation (MTT assay)

Treated cells were spread in 96-well plates at 5000 cells/well, with three multiple wells per well, and cultured for 72 h. Tests were performed for every 12 h of incubation. MTT reagent was added (5 mg/ml prepared with PBS solution) and incubated for 4 h. The supernatant was discarded, 150ul of DMSO was added per well, which was then shaken for 10 min. OD (490 nm) was determined by the enzyme-linked immunoassay and the proliferation curves were drawn (time is abscissa, OD is ordinate).

### Detection of cell invasiveness (Transwell invasiveness experiment)

Matrigel gel was released with PBS buffer solution (1:8) at 4 °C. 100 μL of diluent was applied to the surface of polycarbonate membrane in the upper chamber of Transwell, and placed at 37 °C for 0.5–1 h. Cell suspensions were taken, which was washed with PBS, and the suspended cells were inoculated into the serum-free medium for culture, with the density adjusted to 1–10*10^5^ cells/mL. In the lower chamber of 24-well plate, 500 μL of 5% FBS was added in culture, with Transwell chambers placed inside with forceps. 100 μL of cell suspension was added to the upper chamber and the cells were incubated in a cell culture incubator for 48 h. After the culture, Transwell chambers were taken out, in which the remaining medium was aspirated, and Matrigel gel and the cells inside the upper chamber of Transwell were carefully wiped with a cotton swab. 600 μL of 4% paraformaldehyde was added to a new 24-well plate and fixed in a Transwell chamber for 20–30 min. The fixative was discarded, and then, unbound crystal violet was removed by staining with 0.2% crystal violet for 10 min followed by washes for 3 times with PBS. The upper side of the chamber was gently wiped with a cotton swab, the non-specifically bound dye was removed, and crystal violet was completely eluted with 3% acetic acid after appropriate air drying The eluate was taken, which was determined with the enzyme-linked immunoassay for OD value (570 nm), and the statistics were graphed.

### Detection of cell apoptosis (ELISA)

The 20 μL of samples to be tested was added to the well-coated ELISA reaction plate, and the positive control group was added with 20 μL of histone-DNA complex. And 80 μL of anti-histone antibody and anti-DNA antibody complex was added to both wells. The supernatant was discarded after shaking at 300 r/min for 2 h. 250 μL of buffer was added to wash for 3 times, then 100 μL of ABTS was added. An additional well was prepared to add ABTS as a colorimetric blank control group. After shaking at 250 r/min for 5 min, the OD was determined with a microplate meter (Dual-wavelength colorimetric method, primary wavelength 405 nm, reference wavelength 490 nm), and the apoptosis curve was drawn (time is abscissa, OD is ordinate). The detection kit was from Roche.

### Western blot

After the DLBCL cell lysates were homogenized and centrifuged, the protein supernatant was extracted and the protein levels were determined by BCA. 20 μg of protein loading was taken out for 6% SDS-PAGE, which was then transferred to PVDF membrane. The membrane was sealed with 5% defatted milk powder for 2 h at room temperature. Anti-Akt, anti-p-Akt, anti PIP3, anti-Bax, anti-bcl-2, and anti-cytochrome C (diluted at 1:1000) were added and incubated overnight at 4 °C. After washing, the horseradish peroxide-labeled secondary antibody (diluted at 1:1000) was added and incubated for 1 h at room temperature, colored by the chemiluminescence instrument, and analyzed with ImageJ software. Reagents were all purchased from Abcam, Cambridge, MA, USA.

### Luciferase reporter assay

MET 3'UTR containing the wild-type or mutant sequences of target-binding site of miR-27b were then cloned into the luciferase reporter vector, respectively. They were co-transfected with the miR-27b mimics in DLBCL cells, and then the luciferase activity was determined with the dual-luciferase reporter assay system (PROMEGA).

### Statistical methods

All experiments were repeated independently for more than 3 times, and the continuous variables were expressed as the mean ± standard deviation. The data were analyzed with the Graph Pad Prism 7 software. Shapiro–Wilk test was used to evaluate the normality of continuous variables. The data of multiple groups were compared with Kruskal–Wallis one-way ANOVA and Tukey post hoc test. The Spearman correlation coefficient was used to test the correlation between the levels of continuous variables in different studies. The Spearman correlation coefficient (r) < 0 meant negative correlation between the two variables, and r > 0 meant positive correlation. If the absolute value of r was 0.8–1, then the two variables have a very strong correlation; r was 0.6–0.8 meant strong correlation; r was 0.4–0.6 meant moderately strong correlation; r was 0.2–0.4 meant weak correlation; r was 0–0.2 meant extremely weak or uncorrelated. The data of two groups were compared with the unpaired t-test. P values were calculated bilaterally, P < 0.05 was defined as statistically significant.

## Results

### miR-27b expression in DLBCL cells was down-regulated

**Fig. 1:**
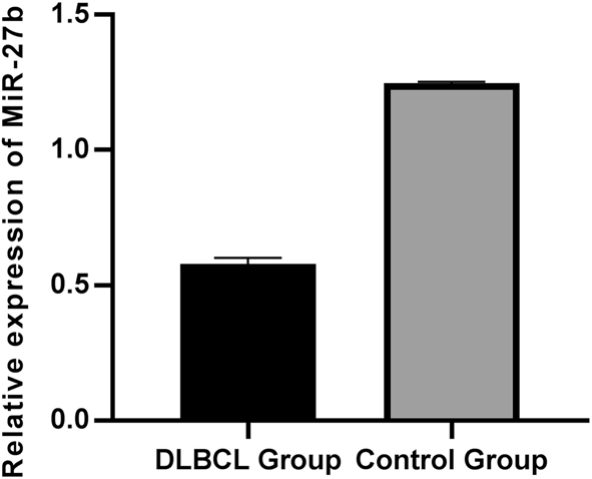
Down-regulation of miR-27b expression in DLBCL cells. According to the comparison of miR-27b expression between disease and control groups, miR-27b expression was significantly lower in the disease group compared with the control group (P < 0.05)

First of all, to clarify miR-27b expression in DLBCL cells and normal human B cell lines, 20 biopsy pathological samples were collected from patients with confirmed DLBCL and miR-27b expression was examined by qRT-PCR. In the meantime, peripheral blood B cell lines from healthy people were matched and also collected as controls at the same time (Table 1) and miR-27b expression was also examined. The results showed that miR-27b expression in DLBCL cells of Diffuse large B-cell lymphoma patients was significantly lower than that in the B cells of peripheral blood in healthy people (Fig. 1), which were statistically significant (P < 0.05).

**Table 1:** Comparison of baseline data between patients with DLBCL and healthy controls

	DLBCL Group (n = 20)	Control Group (n = 20)	*t/χ*^2^value	P value
Male, n(%)	13(65)	12(60)	0.107	0.744
Age, x ± s	58.4(6.61)	57.8(8.62)	0.247	0.806
smoked, n(%)	11(55)	12(60)	0.102	0.749
Family history of cancer, n(%)	2(10)	1(5)	–	1.000
Cardiovascular disease, n(%)	6 (30)	7 (35)	0.114	0.736
Diabetes mellitus, n(%)	4 (20)	5 (25)	–	1.000

### Low miR-27b expression was associated with high proliferation and high invasiveness of DLBCL cells

**Fig. 2:**
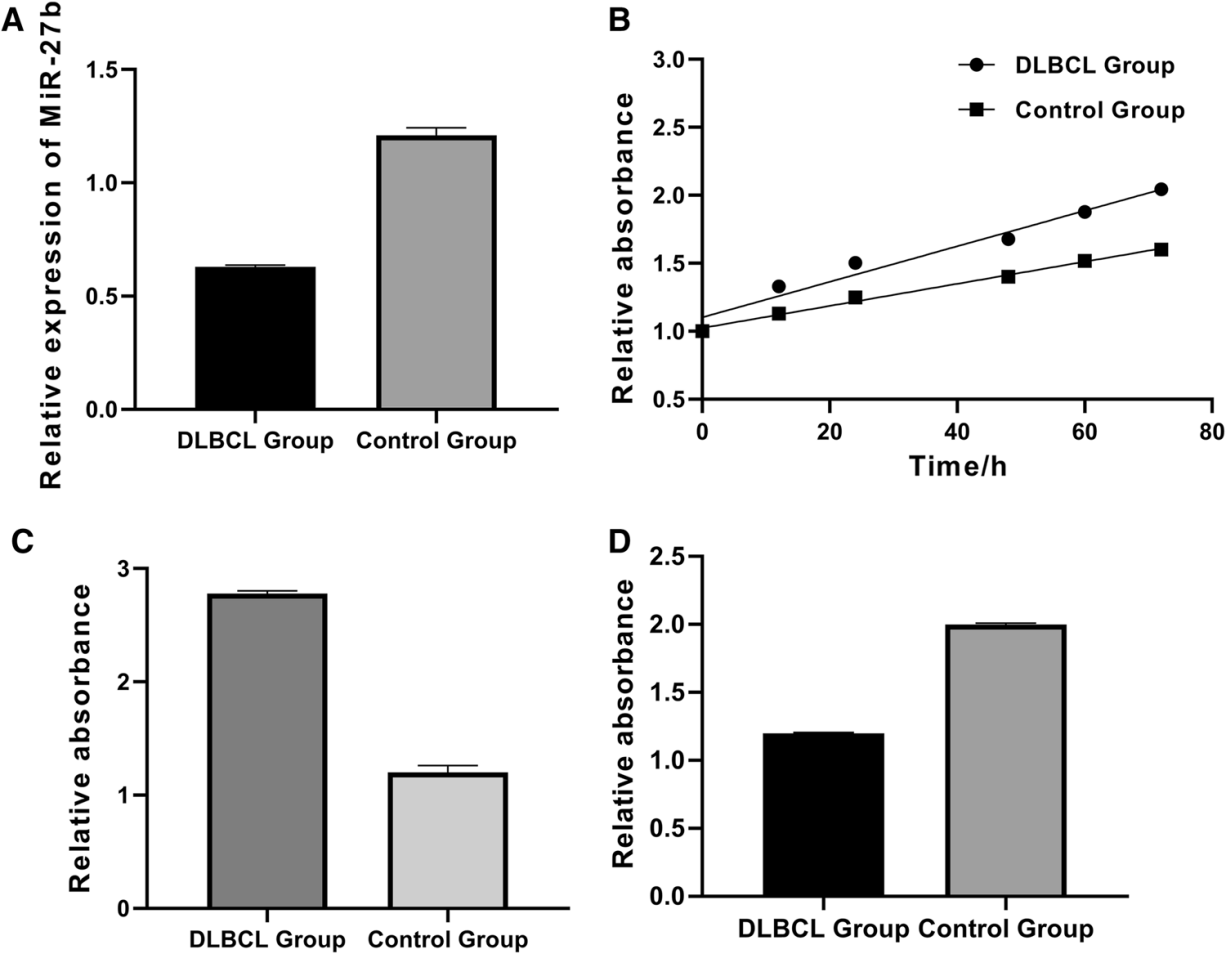
Low miR-27b expression was associated with high proliferation and high invasiveness of DLBCL cells. **A**: miR-27b expressions in two groups were detected by qRT-PCR, and miR-27b expression of DLBCL cells was significantly lower than the control cells (P < 0.05); **B**: The proliferation of both groups was determined by MTT assay, and the proliferation of DLBCL cells was significantly enhanced (P < 0.05); **C**: The invasiveness of both groups was determined by Transwell invasiveness assay, and the invasiveness of DLBCL cells was significantly enhanced (P < 0.05); **D**: The apoptosis of both groups was determined by ELISA, and the apoptosis of DLBCL cells was significantly reduced (P < 0.05)

Human DLBCL cell lines were purchased and miR-27b expression was examined by qRT-PCR. Normal human peripheral blood B cell lines were also purchased as controls and miR-27b expression was also determined by qRT-PCR. The proliferation, invasiveness and apoptosis of cells were determined by MTT assay, Transwell and ELISA, respectively. The results showed that miR-27b expression of DLBCL cells was significantly lower than that in the control group (Fig. 2A). Compared with the control group, the proliferation of DLBCL cells was significantly enhanced (Fig. 2B), with enhanced invasiveness (Fig. 2C), while the apoptosis was significantly reduced (Fig. 2D), which were statistically significant (P < 0.05).

### High miR-27b expression inhibited the activity of DLBCL cells.

**Fig. 3:**
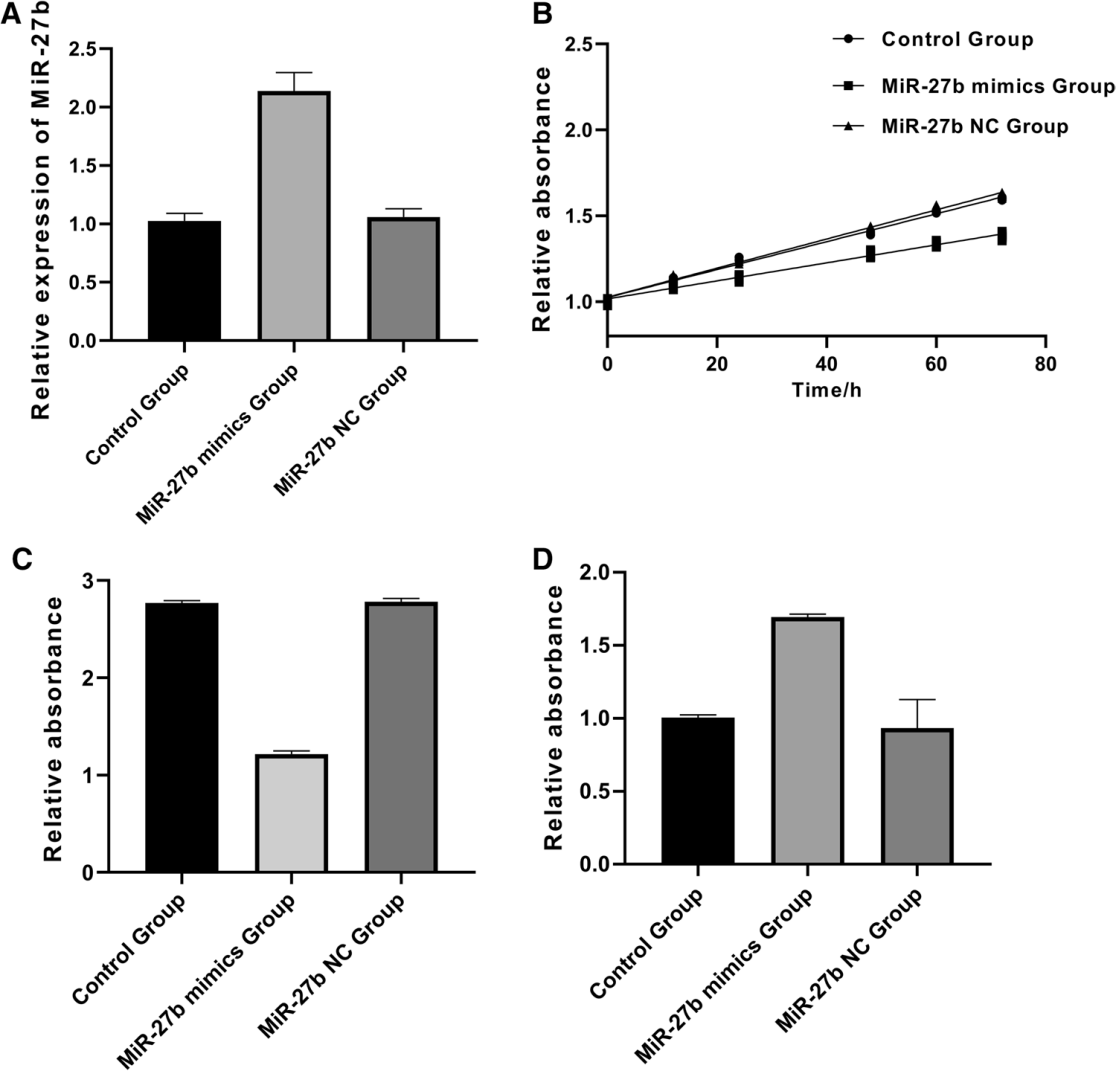
High expression of miR-27b inhibits the activity of DLBCL cells. **A**: miR-27b expression levels in two or three groups were determined by qRT-PCR, and miR-27b expression increased in the miR-27b mimics group (P < 0.05); **B**: The proliferation of the three groups was determined by MTT assay, and the proliferation in the miR-27b mimics group decreased (P < 0.05); **C**: The invasiveness of both groups was determined by Transwell invasiveness assay, and the invasiveness in the miR-27b mimics group decreased (P < 0.05); **D**: The apoptosis in the three groups was determined by ELISA, and the apoptosis in the miR-27b mimics group increased (P < 0.05)

To further clarify the effects of different levels of miR-27b expression on biological functions of DLBCL cells, miR-27b expression in DLBCL cells was over-expressed by cell transfection, and miR-27b expression levels of the three groups were determined by qRT-PCR (Fig. 3A), and the proliferation, invasiveness and apoptosis of the three groups were determined by MTT assay, Transwell and ELISA. The results showed that compared with the miRNA-27b NC group, the proliferation and invasiveness of the miR-27b mimics group significantly decreased (Fig. 3B, C), and the apoptosis significantly increased (Fig. 3D), which showed significant differences (P < 0.05). Therefore, it was speculated that high miR-27b expression may inhibit the proliferation and invasiveness of DLBCL cells and promote the apoptosis of the cells.

### miR-27b directly targeted MET

**Fig. 4:**
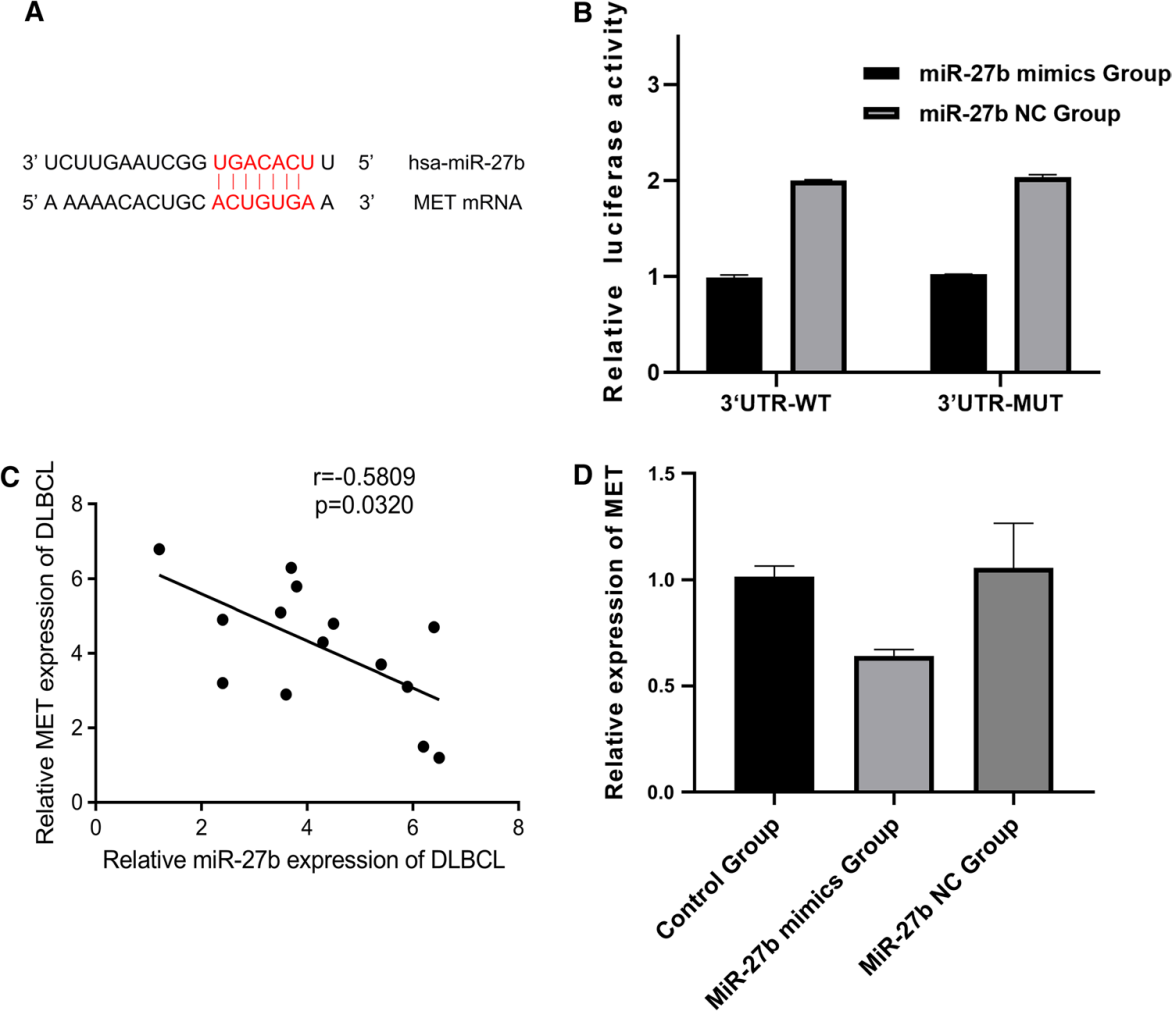
miR-27b directly targeted MET. **A**: The paired sequence of miR-27b and MET; **B**: Dual-luciferase reporter analysis confirmed that miR-27b bound to MET; **C**: the linear regression fitting curve of miR-27b and MET; **D**: MET was poorly expressed in the miR-27b mimics group (P < 0.05)

MET is a proto-oncogene that can participate in the growth cycle, proliferation, migration and invasiveness of cells by regulating the PI3K/AKT signaling pathway. MET has been shown to be actively expressed in a variety of tumor cells, and is closely related to the growth and proliferation of tumor cells (18–20). To explore the molecular mechanism of miR-27b expression on the biological function of DLBCL cells, it was found by querying the Targetscan database that MET may be a downstream target of miR-27b (Fig. 4A). Meanwhile, a dual-luciferase reporter assay was also conducted to verify the direct targeting of miR-27b to regulate the gene expression of MET (Fig. 4B). The Spearman coefficient assay (Table 2, Fig. 4C) showed a significant negative correlation between miR-27b and MET expression in DLBCL (r = − 0.5809, P < 0.05). MET expression levels of blank control, miR-27b mimics and miR-27b NC groups were also determined by qRT-PCR (Fig. 4D). The results showed that MET expression of the miR-27b mimics group was significantly lower than that of blank control and miR-27b NC groups, which was significantly different (P < 0.05).

**Table 2:** Rank correlation coefficient statistics of Spearman

Correlation		miR-27b	MET
Spearman rank correlation coefficient			
miR-27b	Coefficient	1.000	− 0.5809^a^
	Significance (double tail)		0.0320
	N	14	14
MET	Coefficient	− 0.5809^a^	1.000
	Significance (double tail)	0.0320	
	N	14	14

^a^The correlation was significant at the 0.05 level (two-tailed); The absolute value of r was 0.4-0.6, and miR-27b and MET had a moderate correlation.

### miR-27b inhibited the activity of PI3K/AKT pathway

**Fig. 5:**
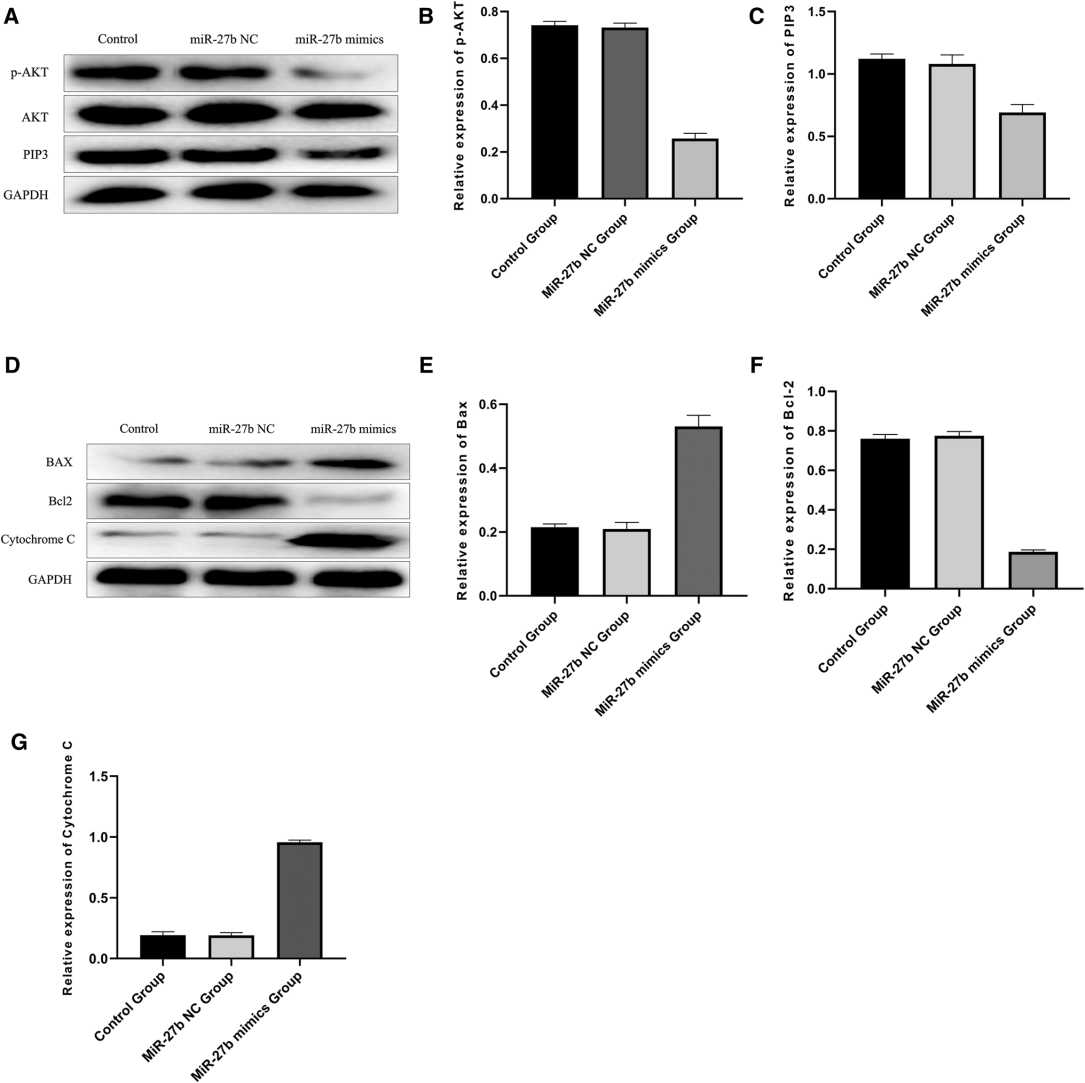
miR-27b inhibited the activity of PI3K/AKT pathway in DLBCL cells. **A**, **C**: The expressions of AKT, p-AKT, PIP3 and GADPH were determined by Western blot. it was thus clear that the expressions of p-AKT and PIP3 in the miR-27b mimics group significantly decreased (P < 0.05); **D**–**G**: The expressions of Bax, Bcl-2 and cytochrome C in three groups were determined by Western blot, and it was clear that Bcl-2 expression in the miR-27b mimics group decreased, while the expression levels of Bax and cytochrome C increased (P < 0.05)

Previous relevant studies have shown that miR-27b can play a biological role by inhibiting the activity of PI3K/AKT pathway [23]. To further validate the molecular mechanism by which miR-27b exerted a biological role, the activation of PI3K/AKT pathway was determined by determining the expressions of AKT, p-AKT, PIP3 and GADPH cells in blank control, miR-27b mimics and miR-27b NC groups by Western blot. The results showed that the expressions of p-AKT and PIP3 significantly decreased in the miR-27b mimics group compared with blank control and miR-27b NC groups (Fig. 5A, C), which were statistically significant (P < 0.05).

In addition, the expression of the apoptosis-related proteins downstream the PI3K/AKT pathway, including Bax, Bcl-2 and cytochrome C, in three groups of DLBCL cells were also examined by Western blot. The results showed lower Bcl-2 expression in the miR-27b mimics group and higher expression of Bax and cytochrome C compared with blank control and miR-27b NC groups (Fig. 5D, G) (P < 0.05). In conclusion, it was speculated that miR-27b may play a role in regulating the proliferation and the apoptosis of DLBCL cells by inhibiting the activity of PI3K/AKT pathway.

### Inhibiting the activation of MET can eliminate the different expression levels of miR-27b on the different effects of the activity of PlI3K/AKT pathway, proliferation and apoptosis of cells.

**Fig. 6:**
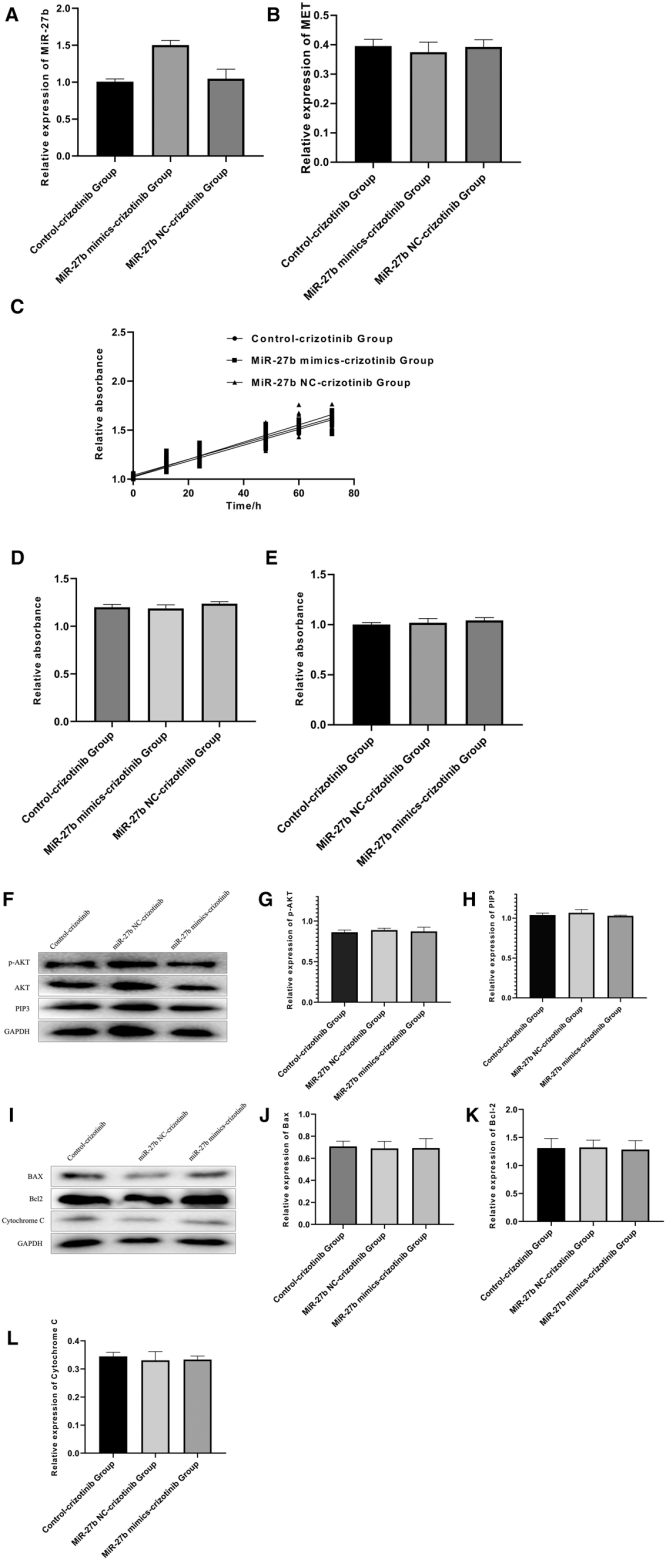
Inhibiting the activation of MET can eliminate the effects of the activity of PI3K/AKT pathway, proliferation and apoptosis of cells. **A**: miR-27b expression was determined by qRT-PCR; **B**: MET expression was determined by qRT-PCR, without statistical difference between groups (P < 0.05); **C**: The proliferation was determined by MTT assay, without statistical difference between groups (P < 0.05), **D**: The invasiveness of cells was determined by Transwell invasiveness assay, without statistical difference between groups (P < 0.05), **E**: The apoptosis was determined by ELISA, without statistical difference between groups (P < 0.05); **F**–**H**: The expressions of AKT, p-AKT and PIP3 in each group were determined by Western blot, without statistical difference between groups (P < 0.05); **I**–**L**: The expressions of Bax, Bcl-2 and cytochrome C were determined by Western blot, without statistical difference between groups (P < 0.05)

To further validate the regulatory role of miR-27b on MET/PI3K/AKT pathway, DLBCL cells in blank control, miR-27b mimics and miR-27b NC groups were treated with high-dose MET inhibitor Crizotinib, and MET gene expression was almost completely inhibited. miR-27b expression level (Fig. 6A), MET expression level (Fig. 6B), proliferation (Fig. 6C), invasiveness (Fig. 6D), apoptosis (Fig. 6E) of cells and the activity of PI3K/AKT pathway (Fig. 6F, L) in the three groups were detected, and compared with those of other three groups without MET inhibitor. The results showed that compared with the non-MET inhibition group, there was almost no difference in the effects of different miR-27b expression levels on the activity of PI3K/AKT pathway, proliferation and apoptosis of cells when MET expression was almost completely inhibited (P > 0.05). Therefore, it was speculated that miR-27b inhibits the proliferation and promotes the apoptosis of DLBCL cells by targeting the expression of MET and inhibiting the activation of MET/PI3K/AKT pathway.

**Fig. 7:**
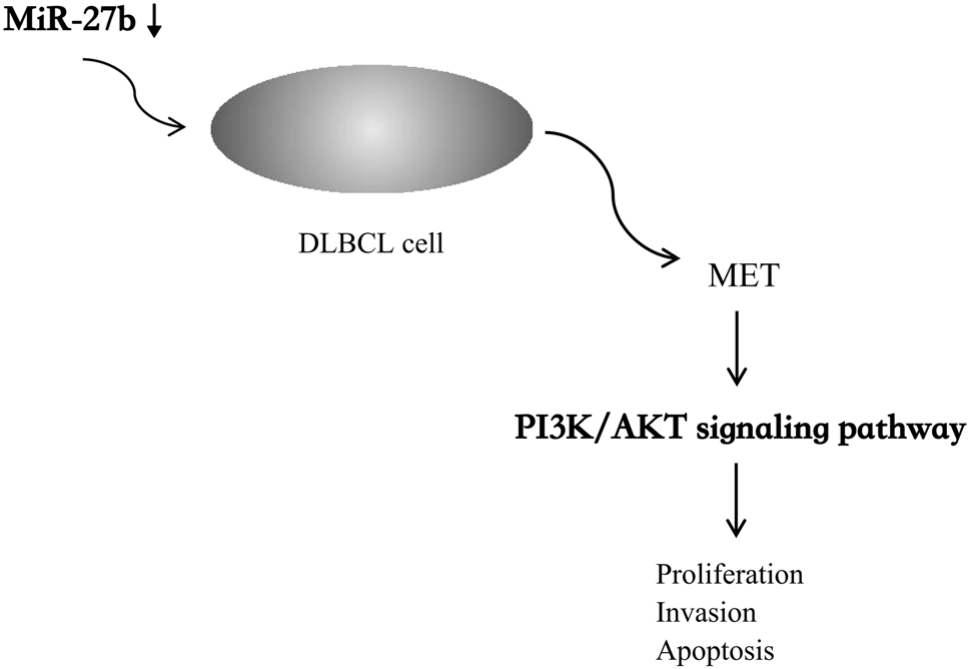
Schematic diagram of miR-27b regulating MET/PI3K/AKT signaling pathway

In conclusion, these results indicate that miR-27b expression in DLBCL cells significantly decreased, while high miR-27b expression inhibited the growth of DLBCL cells and promoted the apoptosis of the cells. miR-27b can specifically inhibit MET and the MET/PI3K/AKT pathway, thus inhibiting the proliferation of DLBCL cells and promoting the apoptosis of the cells, thus inhibiting the progression of tumors (Fig. 7).

## Discussion

DLBCL is the most common type of adult non-Hodgkin lymphoma, and its high invasiveness, high disability and high mortality make it one of the major diseases that endanger people’s life and health. Although the current R-CHOP regimen of Rituximab combined with conventional chemotherapy regimens has been widely used in patients with DLBCL and has significantly improved the long-term survival of such patients, some of them remain refractory after receiving regular complete courses of chemotherapy. Therefore, in order to better identify the traditional treatment of high-risk patients with DLBCL, the researchers developed the international prognostic index, gene expression profile and other risk stratification tools for risk stratification of patients. However, the existing tools cannot fully meet the clinical needs, and the researchers are searching for new biomarkers that can be used in clinical practice to more accurately identify high-risk patients and guide subsequent treatment.

microRNA is a small non-coding RNA, with the main physiological role to act as a post-transcriptional regulator of gene expression to exert its oncogenic or tumor suppressor role. It has been confirmed that microRNA plays an important role in the occurrence and development of multiple neoplastic diseases [12–14]. Therefore, in recent years, more and more researchers have begun to pay attention to and study microRNA, in order to serve as a new predicted therapeutic effect and long-term biomarker for patients with DLBCL. Currently, both miR-155-5p and miR-21-5p are the more frequently studied biomarkers against DLBCL. Several studies have shown that the expression levels of miR-155-5p and miR-21-5p significantly increased in patients with DLBCL [24–27]. And some studies have confirmed that high miR-155-5p expression is associated with the tolerance to chemotherapy and the poor prognosis in patients with DLBCL [28]. However, some literature denies this view [29]. Several other studies on other microRNAs have been reported. Some of the findings suggest that the up-regulation of miR-222-3p expression levels is also associated with a worse prognosis in patients with DLBCL [30, 31]. However, some studies did not observe the correlation between miR-222-3p expression and the prognosis of patients with DLBCL [32]. Furthermore, it has also been shown that the expression levels of miR-18a and miR-181a are predictive primers for the overall survival and progression-free survival in patients with DLBCL receiving chemotherapy with the R-CHOP regimen [31]. It has been indicated that high expressions of miR-28-5p, miR-214-5p, miR-339-3p and miR-5586-5p are associated with a better prognosis in patients with DLBCL, while high miR-324-5p expression often suggests a poor prognosis in such patients [29]. In conclusion, although the mechanism of various microRNAs on DLBCL have been explored and reported, no reliable microRNAs can be used as a prognostic marker for DLBCL. Therefore, the biological functions and specific molecular mechanisms of microRNAs in DLBCL remain to be further studied and clarified.

microRNA-27b is a microRNA with the functions of regulating the proliferation, differentiation and apoptosis of human cells. It regulates the growth cycle and division growth mainly by regulating target genes such as Nischarin, FZD7 and PPARγ. It is often regarded as a tumor suppressor. At present, many studies have shown that miR-27b plays an important biological role in the growth, invasiveness and metastasis of colon cancer, bladder cancer, renal cell cancer and lung cancer. Its low expression in tumor cells is closely related to the proliferation and high invasiveness of tumor cells [22, 33–36]. The expression of microRNA-27b is regulated by a variety of factors. For example, 1,25(OH)_2_D_3_ inhibits TGF-β1-induced lung fibroblast differentiation by targeting miR-27b37; Liraglutide regulates the expression of miRNA-27b in serum [38], while miR-27b-SP acts as a lipid enhancer by directly increasing the expression of adipogenic transcription factors, thereby increasing lipogenesis [39]. Recently, some literature have reported that miR-27b may play an important role in the development of DLBCL [17]. However, its clinical significance and potential mechanism in this process are not completely clear and need to be explored. This study aims to provide a theoretical basis for finding new biomarkers to identify high-risk patients by exploring the influence of miR-27b expression on the biological functions of DLBCL cells and its specific molecular mechanisms, and also to provide some ideas and references for finding new therapeutic targets in the future.

MET, a proto-oncogene located on chromosome 7q21-q31, exerts its functions in regulating the growth cycle, proliferation, migration and invasiveness of cells by regulating the signaling pathways such as PI3K/AKT pathway, RAS/ERK/MAPK pathway and FAK pathway. At present, studies have confirmed that MET expression is active in various tumor cells, such as lung cancer and glioma, and is closely related to the growth and proliferation of tumor cells. The migration and promotion of cell survival by MET activation-induced cell dynamics is the basis for the mechanisms of invasiveness, metastasis and drug resistance of tumor cells [13–15]. Therefore, some researchers have developed targeted treatment programs for MET genes in recent years, and it has been proven to be effective in some tumors showing met dependent status [40].

Phosphatidylinositol 3-kinase (PI3K)/AKT signaling pathway is one of the major signaling pathways for cells that play an important role in basic intracellular functions, which also plays an important role in regulating the proliferation, growth, size, metabolism and motility of cells. PI3K/AKT pathway can exert anti-apoptotic effects by modulating the activity of Bcl-2 family. It has been demonstrated in several studies that PI3K/AKT pathway is activated in a variety of cancers in humans, while inhibiting the activity of PI3K/AKT pathway suppresses the proliferation of tumor cells, and it has been investigated in several clinical trials [21].

To explore the effect of miR-27b on the biological functions of DLBCL cells, we determined the miR-27b expression levels in human DLBCL cell lines and normal human peripheral blood B cell lines by qRT-PCR, and compared the proliferation and apoptosis of the two groups. The results showed that miR-27b expression in DLBCL cells decreased significantly and correlated with their enhanced cellular activity. We over-expressed miR-27b in DLBCL cells and examined its biological functions. The results showed that high miR-27b expression inhibited the proliferation of DLBCL cells and increased the apoptosis of the cells. We determined the activation of PI3K/AKT pathway in DLBCL cells with different miR-27b expressions, and found a significant inhibition of the activation of PI3K/AKT pathway in DLBCL cells with miR-27b over-expression. Inhibition of MET expression by high-dose Crizotinib can eliminate this effect. According to the results in Fig. 6B, the inhibitory effect of crizotinib on EMT was almost the same with or without miR-27b NC, but with miR-27b mimics, the EMT expression can be reduced. Therefore, in the effect of miR-27b acting on EMT/PI3K/AKT pathway on diffuse large B-cell lymphoma, crizotinib can achieve the same effect as miR-27b NC to activate the expression of miR-27b to achieve the inhibition effect of EMT. And by inhibiting the activation of the MET/PI3K/AKT pathway, the proliferation ability of diffuse large B-cell lymphoma cells was inhibited and their apoptosis was promoted. Crizotinib may also be a suitable drug to increase the sensitivity of DLBCL cells to R-CHOP treatment, so in addition to the R-CHOP regimen, crizotinib/EMT pathway will likely be a new idea for the treatment of non-Hodgkin lymphoma.

In conclusion, our study showed that miR-27b expression significantly decreased in DLBCL cells, while high miR-27b expression inhibited the growth of DLBCL cells and promoted the apoptosis of the cells. miR-27b could inhibit the proliferation of DLBCL cells and promote the apoptosis of the cells by targeting MET and inhibiting the MET/PI3K/AKT pathway, thus inhibiting tumor progression. By exploring the role of miRNA-27b in the occurrence and development of DLBCL, we further defined the specific molecular mechanisms during the proliferation and invasiveness of tumor cells, providing a reference for finding new biomarkers to identify high-risk patients with DLBCL who have poor conventional treatment results. With the further progress and the further improvement of mechanisms in the proliferation and invasiveness of tumor cells, we may find the key target of mechanisms in the proliferation and invasiveness of tumor cells, and explore new treatment regimens for it. Our goal is to achieve better therapeutic outcomes by combating tumor cells with higher precision, while reducing side effects during treatments and improving the quality of life and long-term prognosis of patients with DLBCL.

## Data Availability

The datasets used and/or analyzed during the current study are available from the corresponding author on reasonable request.
